# A Multi-Faceted Strategy for Evidence Translation Reduces Healthcare Waiting Time: A Mixed Methods Study Using the RE-AIM Framework

**DOI:** 10.3389/fresc.2021.638602

**Published:** 2021-03-23

**Authors:** Katherine E. Harding, Annie K. Lewis, David A. Snowdon, Bridie Kent, Nicholas F. Taylor

**Affiliations:** ^1^Allied Health Clinical Research Office, Eastern Health, Melbourne, VIC, Australia; ^2^School of Allied Health, La Trobe University, Melbourne, VIC, Australia; ^3^Faculty of Health, University of Plymouth, Plymouth, United Kingdom

**Keywords:** waiting, waiting and queuing, REAIM, implementation science, research translation, scheduling, community, outpatient

## Abstract

**Background:** Waiting lists are often thought to be inevitable in healthcare, but strategies that address patient flow by reducing complexity, combining triage with initial management, and/or actively managing the relationship between supply and demand can work. One such model, Specific Timely Appointments for Triage (STAT), brings these elements together and has been found in multiple trials to reduce waiting times by 30–40%. The next challenge is to translate this knowledge into practice.

**Method:** A multi-faceted knowledge translation strategy, including workshops, resources, dissemination of research findings and a community of practice (CoP) was implemented. A mixed methods evaluation of the strategy was conducted based on the RE-AIM (Reach, Effectiveness, Adoption, Implementation, and Maintenance) framework, drawing on an internal database and a survey of workshop and CoP participants.

**Results:** Demonstrating reach, at July 2020 an internal database held details of 342 clinicians and managers from 64 health services who had participated in the workshop program (*n* = 308) and/or elected to join an online CoP (*n* = 227). 40 of 69 (58%) respondents to a survey of this population reported they had adopted the model, with some providing data demonstrating that the STAT model had been efficacious in reducing waiting time. Perceived barriers to implementation included an overwhelming existing waiting list, an imbalance between supply and demand and lack of resources.

**Conclusion:** There is high quality evidence from trials that STAT reduces waiting time. Using the RE-AIM framework, this evaluation of a translation strategy demonstrates uptake of evidence to reduce waiting time in health services.

## Introduction

Waiting for healthcare services is a perennial problem in healthcare, and non-emergency services provided in community and outpatient settings are particularly susceptible to the development of lengthy waitlists. When there is a perception that patients can wait, they often do, with prioritization systems commonly used to try to ensure that at least those with urgent needs can access timely care (1). Services provided in community and outpatient settings include allied health services, rehabilitation, chronic disease management programs and a broad variety of healthcare services provided through community health services. Delays in access to these services have consequences, including lower levels of engagement, missed opportunities for treatment, and worse health outcomes (2–4). Furthermore, long waiting lists contribute to service inefficiencies, as resources are redirected from frontline care to managing the waiting list (5).

Waiting lists for these services are not inevitable. There is a growing body of evidence that suggests that access can be improved through patient flow initiatives that balance supply and demand, reduce service inefficiencies, and provide rapid access to early assessment. Coupled with short-term initiatives to reduce the existing backlog of waiting patients, these approaches can lead to sustainable reductions in waiting time (6–8). One such model, known as Specific Timely Appointments for Triage (STAT), brings together these evidence-based principles and presents them in a structured, step-by-step process that can be readily implemented by service providers (9). STAT is based on two key principles: the creation of protected new appointments calculated from analysis of demand, alongside a one-off intervention to manage and reduce or eliminate the existing wait list. A feature of this approach is that, without a significant wait list, each patient can be offered the next available appointment and be seen in a timely way, negating the need for complex prioritization systems or other processes to manage a waiting list. Priority decisions take place after the initial assessment, and are focussed on the need for follow up or review appointments rather than initial access to the service. These decisions are made by clinicians who have a complete understanding of the patient's situation within the context of competing service demands (10).

There is high quality, published evidence showing that the STAT model is effective in reducing waiting time (9, 11, 12). The model was initially tested in a controlled before and after trial in a community rehabilitation program, reducing waiting time by 40% at the intervention site, with no significant change at the control site (9). A second trial applied STAT to an outpatient physiotherapy service, and measured a mean reduction in waiting time of 20%. Waiting time for patients at the 75th percentile reduced from 33 to 21 days, suggesting that the model had the greatest impact on those previously waiting the longest (11). The largest trial of STAT was completed using a stepped-wedge cluster randomized controlled trial design involving eight community outpatient services and 3,113 participants. After implementing STAT, a 34% reduction in waiting time could be attributed to the intervention after controlling for clustering by service (12). In all three trials, STAT also led to large reductions in variability in waiting time. In a follow up study of the stepped wedge trial, waiting time reductions were still observed at 12 months (13).

However, it is well-established that generating evidence and publishing research is not sufficient to bring about a change in practice (14). Research translation is a process of knowledge generation and transfer that enables the knowledge to be applied in practice. Effective research translation leads to research impact; demonstrable improvements to human health and society (15). A range of strategies have been shown to be effective in the implementation of evidence, but multi-faceted interventions have a higher level of impact than single interventions (16). Therefore, in order to translate the findings of evaluations of the STAT model into practice beyond research trials, a suite of research translation activities was designed to facilitate uptake and implementation of the intervention into clinical practice.

RE-AIM is a framework for evaluating and reporting on the translation of evidence into practice. It was designed to “improve the sustainable adoption and implementation of effective, generalizable, evidence-based interventions” (17). The framework includes the five domains of reach, effectiveness, adoption, implementation and maintenance and provides an ideal structure in which to evaluate the effectiveness of efforts to translate evidence into practice. RE-AIM has become one of the most widely used implementation frameworks, and has been applied across a broad spectrum of health settings and populations, and many countries and cultures (18, 19).

We used the RE-AIM framework to evaluate the outcomes of a multi-faceted strategy to facilitate translation of research evidence of the effectiveness of the STAT model into practice, and to identify early indications of research impact in reducing health service waiting times.

## Methods

### Setting

The STAT model was developed and tested at Eastern Health, a large metropolitan health network in the eastern suburbs of Melbourne in collaboration with La Trobe University. A research translation strategy was designed to translate the research findings to providers of publicly funded outpatient and community health services primarily across the state of Victoria, Australia. Victoria has a population of 6.6 million people, of whom almost 5 million reside in the capital city of Melbourne and surrounding suburbs. The remainder lives across regional centers and rural areas. A small number of people from outside Victoria took part in one or more activities and were eligible to participate in this study.

The targets for this research translation strategy were clinicians and managers working in publicly funded community rehabilitation programs (typically providing rehabilitation for new onset conditions or following hospital discharge), community health services (typically providing care for ongoing disability or chronic conditions), allied health hospital outpatient services (usually single discipline services for acute or post-surgical interventions), and multi-disciplinary specialist clinics (specialist care from multi-disciplinary teams for conditions such as dementia, falls, continence, or movement disorders). In Victoria, community rehabilitation, outpatient allied health and specialist clinics are usually organized under the auspices of large metropolitan health networks or regional district health services. Community health services can be associated with larger health networks but are more often run as independent organizations serving a local community.

### Components of the Research Translation Strategy

The research translation strategy included four major components: A series of training workshops; development of freely available resources; an email based community of practice (CoP); and traditional dissemination of research outputs through publications and presentations. The center-piece of the research translation strategy was a half-day workshop designed to develop opinion leaders who could become “product champions” within their services (20). The workshop aimed to provide participants with an understanding of the theory and evidence supporting the STAT model, and a practical, stepwise approach to implementing the model in their health services. It was designed to be delivered to groups of up to 40 people, in different locations around the state. Two members of the research team involved in developing and evaluating the model delivered the workshops using a combination of didactic presentations and small group activities, as supported by literature on changing behavior of healthcare workers (21, 22). A pilot workshop was held in December 2017 at a single health network, and the first open workshop was run in June 2018. Subsequent workshops were scheduled based on demand, often prompted by requests from health care providers. Workshops were advertised using email and social media circulation through leadership groups of major providers of public health services.

Resources included a 30-page handbook and a 3-min, animated video to explain how the model works. A hard copy of the handbook was provided to workshop participants, but the video and handbook were also made freely available online (23). Workshop participants or others accessing the resources were invited to contact the researchers if they had questions or required further information during the implementation process.

People who expressed interest in the model either by attending a workshop or contacting the research team were given the opportunity to join a CoP. The term “community of practice” is not always clearly defined in healthcare, but was originally proposed by Wenger to be a group of people sharing the three dimensions of a shared purpose, mutual interactions, and a shared repertoire of resources, tools and knowledge (24). Participants in the STAT CoP had a shared concern about their health service waiting times, a shared training experience and access to common resources. The group maintained a connection primarily through distribution of emails, updating participants on news and training opportunities related to the STAT model, and offering a point of contact for people needing further information or support with implementation.

Research findings were also disseminated through traditional, academic channels, including six peer-reviewed journals publications and one book chapter (8, 11–13, 25–27) and 16 presentations at conferences and professional events.

### Evaluation Design and Data Sources

The RE-AIM (reach, effectiveness, adoption, implementation, and maintenance) framework was used to guide evaluation of the knowledge translation strategy (18). Data were collected from two sources to inform each of the elements (Table 1): (1) internally collected data from workshop attendance lists and CoP mailing list; (2) a cross-sectional survey of participants in the workshop series and service providers who requested to join the CoP mailing list.

**Table 1:** Summary of outcome measures and data sources organized by domain of the RE-AIM framework.

**Domain**	**Outcome measure**	**Data source**
Reach	The number of individual workshop participants	Internal database
	The number of healthcare organizations represented at the workshops	Internal database
	The number of people who elected to join the community of practice	Internal database
	Characteristics of survey respondents	Survey
Effectiveness	Evidence provided by survey respondents who implemented STAT of change in waiting times at their services.	Survey
Adoption	The number of survey respondents who reported implementing the model at their service	Survey
	The number workshop participants who would recommend it to others.	Survey
Implementation	Barriers to implementation	Survey
	Factors perceived to be associated with success	Survey
	Value of different translation strategies to support implementation	Survey
Maintenance	Comments from survey respondents indicative of the STAT model having become embedded in practice	Survey
	Comments regarding sustainability of waiting time reductions	Survey

All participants who attended the workshops and people on the CoP mailing list were sent an email with an invitation to complete a short online survey. The survey was distributed on two occasions; initially in July 2019 and then again in July 2020 to people who had subsequently attended a workshop or been added to the mailing list. It was common for health organizations to have several representatives attend a workshop together. Participants were invited to participate in the follow-up survey as individuals but it was expected that some would elect to have a single representative complete the survey on behalf of their team and that this would impact on the response rate.

The survey questions were designed specifically for the study and included questions regarding: (1) participants' views of the training and associated resources; (2) any subsequent actions taken at their workplace to implement the STAT model; (3) observed impact of these actions at their service; (4) factors that were barriers or facilitators to implementation. Given that future implementation of the STAT model is likely to be affected by the experience of early adopters and the degree to which they influence others, participants were also asked to state on a scale of 1–10 how likely they would be to recommend the STAT model to other services. This enabled calculation of a Net Promoter Score (NPS); a calculation of the difference between the percentage of “promoters” (people who give a rating of 9 or 10) and “detractors” (people who give a rating of 1–6), which has been linked with growth in service industries and been used for measuring consumer satisfaction with healthcare (28, 29). There is little evidence available to judge what a “good” NPS may be for adoption of a health service innovation, but in the service industries, any score above zero (indicating more promoters than detractors) is generally considered to be positive, and a score above 50 is considered excellent (28).

Data were analyzed descriptively, and presented using frequencies. Chi square statistics were used to explore differences between survey respondents and non-respondents, and between survey respondents who had implemented the STAT model and those who had not. Where a variable had more than two categories and differences were significant, *post-hoc* tests were conducted using adjusted residuals with Bonferroni adjustment to identify the contributing cells (30).

## Results

### Characteristics of Survey Sample

Invitations to participate in the survey were sent to 342 people in an internal database who had either participated in a workshop or independently made contact with the research team. Of these, an estimated 300 emails reached the intended recipients. Seventy-three individuals responded to the survey, a response rate of 24%. At least one response was received from 35 healthcare organizations, equating to a response rate of 54% at the service level.

Comparing characteristics of survey respondents and non-respondents, both groups were similar in relation to geographical location (regional or metropolitan) and the type of organization they worked for. Compared to non-responders, more survey participants were in senior clinician positions and fewer were mid-level clinicians, and more had participated in a workshop and elected to join the CoP (Table 2).

**Table 2:** Characteristics of survey respondents and potentially eligible participants from the distribution list.

	**Survey respondents**	**Non-respondents**	**Total eligible population**	**Difference between responders/** **non-responders(Chi Square)**
*n*	73	269	342	
Location [*n*(%)]				
Metropolitan	36 (49)	140 (52)	176 (53)	*P* = 0.68
Rural	37 (51)	129 (48)	166 (49)	
Position [*n*(%)]^**^				
Manager	24 (33)	85 (40)	109 (39)	*P* < 0.01
Senior clinician/Team leader	36 (49)^*^	57 (27)^*^	93 (33)	
Clinician	8 (11)^*^	50 (24)^*^	58 (21)	
Admin/Intake	2 (3)	13 (6)	15 (5)	
Other	2 (3)	6 (3)	8 (3)	
Attended workshop				
Yes	71 (97)	237 (88)	308 (90)	*P* = 0.02
No	2 (3)	32 (12)	34 (10)	
Type of organization				
Large metro health network	27 (37)	112 (42)	139 (41)	*P* = 0.85
Regional health network	32 (44)	102 (38)	134 (39)	
Community health center	12 (16)	12 (16)	58 (17)	
Government (local or state)	2 (3)	7 (3)	9 (3)	
University	0 (0)	2 (1)	2 (1)	
Elected to join COP mailing list				
Yes	55 (75)	172 (64)	227 (67)	*P* < 0.01
No	18 (25)	97 (36)	115 (34)	

* *Source of difference between cells using post-hoc testing using adjusted residuals with Bonferroni adjustment.*

** *No data available on Position for 58 non-respondents and 1 respondent*.

Results are presented using each of the elements of the RE-AIM framework. Data on reach and adoption are presented first, followed by barriers and facilitators to implementation, and finally effectiveness and maintenance of the STAT model are discussed in relation to the services that adopted the model.

### Reach

The half-day workshop was delivered on 12 separate occasions. Eight took place in metropolitan Melbourne and three in regional Victoria. One additional workshop was held in Hamilton, New Zealand. In total, the workshops reached 308 participants from 63 healthcare organizations, including 13 large metropolitan health networks (incorporating a broad range of hospital and community services), 24 regional health services (incorporating at least one regional hospital and associated outpatient services), and 20 community health services. A small number of government representatives also participated.

At July 2020, 227 people had requested to join the CoP, from 61 different healthcare organizations. Most had attended workshops, but the CoP also included a small number of people who had contacted the researchers after reading an academic publications or attending a conference presentation.

### Adoption

Forty respondents (56%) to the survey reported that they had either implemented or were in the process of implementing STAT at their service. The differences in distributions across service type did not reach statistical significance (χ^2^ = 4.3(*df* = 2), *p* = 0.12), but community rehabilitation services appeared most likely to adopt the model, with 11 of 15 respondents from these services having either implemented STAT, or being in the process of implementation. The majority of respondents from community health centers (61%) also reported adopting the model. The remaining respondents were from a mixed group of services including outpatient allied health and specialist clinics with diverse models of care. Of these, 7 (42%) reported adopting STAT (Table 3).

**Table 3:** Survey participant responses to the question “Have you implemented the STAT model at your service?” by type of service.

	**Yes/in process *n* (%)**	**No *n* (%)**	**Significance**
			**(Chi square)**
Community Health	22 (61)	14 (39)	*P* = 0.12^**^
Community Rehab	11 (73)	4 (17)	
Other	7 (42)	11 (58)	
Total^*^	40 (58)	29 (42)	

* *No data about adoption provided by 4 respondents.*

** *Omnibus test showed no significant difference in distribution of the data, and therefore no post-hoc analysis performed*.

Participants in the survey were asked to select which of four statements best described their attitude to the STAT model. This question was intended to provide an insight into how people felt about adopting STAT, regardless of whether they had actually been able to do so. Thirty respondents (41%) reported that they had “enthusiastically embraced STAT,” and 13 (18%) selected the option “I think STAT could work in our service.” There were 25 (34%) respondents who felt that the “STAT had value but we face significant barriers to implementation” and 4 (5%) who responded that “STAT is not for us.”

### Effectiveness

Responses to the survey provide preliminary evidence of effectiveness in the health services that implemented the STAT model. Ten respondents to the survey who had implemented STAT provided made non-specific observations about reduced waiting times, and seven gave specific examples of changes observed. Although these data can be considered uncontrolled and anecdotal, collectively the responses provide evidence of changes observed after introducing STAT that is consistent with findings from previous trials. For example:

“*Waiting times now reduced to 2–4 weeks for initial assessment. It was* ~*6–12 months for priority 3's previously” (Regional community health service)*

“*Change in wait-time - average 18 days declined to 6 days” (Metro community rehabilitation program)*

“*Referral to acceptance wait-times reduced by 28 days (95%CI 23.8–32.2, down 74%) and referral to first face to face appointment wait times reduced by 33 days (95%CI 10.5–55.5, 28% reduction)” (Metropolitan pain clinic)*

The remaining respondents who reported implementing STAT did not provide any information about effectiveness, or stated that data were not yet available. No respondent reported that waiting time had either remained unchanged or increased after implementing the model.

### Implementation

Survey data also provided insights into challenges and facilitators for implementing STAT outside research trial settings. The most common challenges, each reported by about a third of respondents, related to large existing waiting lists, a lack of resources, and a perception of a true imbalance between supply and demand. It is not clear from the available data to what extent perceptions reflected reality, but perceptions in themselves are powerful and can be a barrier to further action. Support from management was the most commonly reported factor facilitating implementation, followed by support of staff and organizational culture. Four respondents stated that additional resources had been made available to support implementation.

Survey participants were asked to rate the extent to which they had used or valued the various resources made available to them to assist with implementation. All of the resources were more often reported to be valued by those who had or were in the process of implementing the model (the “implementers”) compared to the “non-implementers” but the patterns of usage were similar across all survey participants (Table 4). The face-to-face workshop training and associated handbook were the most highly valued resources, valued by more than 90% of participants. In contrast, the CoP, individual consultations with the research team and academic publications were each used and/or valued by just over half of survey respondents.

**Table 4:** Use of implementation resources by survey respondents who had and had not adopted the STAT model.

	**Implementers (*n* = 40)**	**Non-implementers(*n* = 29)**	**Total** **(*n* = 73)**^**#**^
Face-to-face training workshops [*n*(%)]			
High value	35 (88)	11 (38)	49 (67)
Moderate value	3 (7.5)	15 (52)	19 (26)
No value/not used	2 (5.0)	3 (10)	5 (7)
Community of practice [*n*(%)]			
High value	5 (13)	5 (17)	11 (15)
Moderate value	16 (40)	9 (31)	27 (37)
No value/not used	19 (48)	15 (52)	35 (48)
Handbook [*n*(%)]			
High value	30 (75)	13 (45)	46 (63)
Moderate value	7 (18)	12 (41)	20 (27)
No value/not used	3 (8)	4 (14)	7 (10)
Consultations [*n*(%)]			
High value	14 (35)	3 (10)	19 (26)
Moderate value	10 (25)	7 (24)	18 (25)
No value/not used	16 (40)	19 (66)	36 (49)
Publications [*n*(%)]			
High value	13 (33)	3 (10)	17 (23)
Moderate value	11 (28)	10 (35)	22 (30)
No value/not used	16 (40)	16 (55)	34 (47)

# *No information regarding adoption provided by 4 survey respondents*.

Of the 68 respondents who offered a response to the question “on a scale of 1 to 10 how likely they would be to recommend the model to a colleague at another service?” 24 (35%) were considered promoters (a score of 9 or 10) and 27 (40%) were considered passives (a score of 7 or 8). Eighteen (26%) were considered detractors (a score of 6 or less), resulting in an NPS of 9. Of the 38 respondents who reported they had implemented the model and answered this question, there were 23 promoters (61%), 15 passives (49%), and no detractors (NPS = 61).

### Maintenance

Participants in the survey responded within 3–12 months after attending the STAT training, and survey data were therefore of limited value in contributing to the question of maintenance. However, of those who had implemented the model, there were no reports of experiences of initial success followed by a rapid return to previous waiting times.

## Discussion

The STAT model was designed to provide a structured, evidence-based approach to help providers of outpatient and community-based healthcare services to reduce long waiting times and improve access for their patients. The model has been shown to be effective in several trials and findings have been disseminated through academic publications and professional conferences, but this does not guarantee that this evidence will be translated into practice or have an impact on service delivery or human health (31). In the current study, about half of participants who were reached by a translation strategy and responded to a survey had adopted the STAT model at their service. While the likelihood of a degree of response bias is acknowledged, these data provide a clear indication that the results of trials of STAT can be replicated in real world settings. This paper adds to existing evidence of effectiveness from trial settings, and makes an important contribution by reporting on translation of the STAT model into practice, including early indications of research impact.

The RE-AIM framework, incorporating the domains of reach, effectiveness, adoption, implementation and maintenance, provides a useful structure in which to view the components and outcomes of this translation strategy (18). The findings of this study can also be interpreted through the lens of diffusion theory. Diffusion is the process through which innovations spread through a social system (32). This process begins with innovators (who, in this case, could be considered the researchers who developed and tested STAT at trial sites), followed by early adopters who are usually motivated by the attributes of the innovation (32). The “implementers” in the current study could be considered in this category. The next phase is adoption by the large majority, who are largely influenced by what others have done before them and a belief that it is the right thing to do. Broader uptake of the STAT model is therefore likely to be influenced by the degree to which the current early adopters influence others. The net promoter score calculated from the question “how likely would you be to recommend STAT to other service providers?” suggests that there is potential for diffusion. An NPS of 9 among all survey responders suggests some limited potential for promotion, but the finding that the NPS was 61 among the early adopters is more encouraging and may contribute to ongoing diffusion of the STAT model.

Diffusion theory can also help to explain why STAT may have been implemented at some sites but not others. Rogers (33) argues that potential adopters are influenced by an innovation's relative advantage, simplicity, and compatibility with existing structures (33). The complexity of implementing STAT with a long existing waiting list was often cited as a perceived barrier; other respondents commented on benefits of reducing waiting time suggesting a perception of relative advantage and the ability to mount a case for change. Some survey respondents expressed reasons why the model was not considered to be a “good fit” for their service. These findings were all consistent with data from qualitative studies of staff experience of implementation of STAT (26, 27). Other elements of the model consistent with diffusion theory that may have supported uptake include the existence of demonstration projects from multiple sites across three trials, and clustering of several elements (including balancing supply and demand, reducing the backlog, efficiency measures and conducting triage at the point of service delivery) into a single intervention (33).

This research translation approach did not rely on a single intervention, but, consistent with other studies reporting success in research translation, used a suite of strategies to try to provide service providers with the skills and knowledge required to implement the model (14, 34). Hands-on training resources were most high valued, and consistent with previous literature, survey respondents were less likely to report they had used or valued the academic publications (35, 36). Both the CoP and follow up consultations were also less widely used and valued strategy, although both were still highly appreciated by some survey respondents. It may be the case that the value of strategies that facilitate interaction with others is influenced by factors such personal preference and alternative sources of support. In addition, the STAT CoP operated at a very basic level with communication driven mainly by the researchers. It is possible that additional benefits could be achieved in this area with more dedicated resources, promotion and opportunities for ongoing interaction between members (37). At an organizational level, services that successfully implemented the STAT model may have benefitted from a form of knowledge brokering. Knowledge brokers act as an intermediary between researchers and decision makers (38). Those who attended the STAT training workshops may have informally acted in this role when they brought the learnings back to their organization, disseminating knowledge and building capacity within their teams to make evidence-based changes to service delivery (39).

Waiting and access delays are well-recognized and persistent problems in healthcare despite the existence of evidence syntheses that demonstrate evidence-based strategies that can be useful in addressing the issue (40). There are many examples in the literature of interventions in outpatient settings at single sites that have led to reductions in waiting time, but they often rely on uncontrolled designs and lack external validity (41). Some examples of approaches to waiting time that have been applied beyond single settings exist, but they have been limited to specific types of services such as general practice (42) or child and adolescent mental health services (43). In contrast, there is a body of evidence, from research trials and the translation activities described in this study, that STAT can reduce waiting time that in a diverse range of services including community health, outpatient allied health services, rehabilitation, and multi-disciplinary specialist clinics across metropolitan and regional areas. The onset of the COVID-19 pandemic in 2020 led to further workshops (conducted after the conclusion of the current study) being delivered online. This forced experiment has enabled participation from service providers across Australia, and paves the way for further scale-up. Future research could explore whether the impact of this method of training is comparable with face to face training. Given that delays in access to care are associated with reduced health outcomes, anxiety, workforce participation and health service costs (2–4), broad scale application of the STAT model has the potential to have an impact on physical and psychological health for millions of people.

This study has some limitations that need to be considered. The individual response rate to the survey was relatively low, although was higher when considered at the organizational level. Data available on the CoP and workshop attendees were also limited, but combined these provide some indication that survey respondents were a representative sample. Data collection was limited to two sources; there are a range of other measures of engagement that could have been collected, such as the number of people who interacted with the researchers and/or CoP outside of the workshops or the number of attendees at conference presentations that may have provided additional insights in relation to reach and implementation. The timeframe between workshop attendance and survey distribution was relatively short for implementation of a service level intervention and is insufficient to inform about maintenance. It is possible that some survey respondents may not have had time to commence implementation, and those who were experiencing success may still face challenges with sustainability. Follow-up over a longer period in future studies will provide further insights into these issues. Strengths of the study were the use of multiple data sources enabling triangulation of results, and that evaluation of the translation strategy was considered within an established framework.

## Conclusion

The STAT model has demonstrated effectiveness by reducing waiting time in multiple trials. This study shows that a multi-faceted research translation strategy has reached services across a large geographical area and been adopted by multiple services and implemented into community health and rehabilitation settings. The study provides insights into factors that have facilitated implementation, and highlights challenges for consideration in future research and translation. The study adds to previous published literature on the STAT model by demonstrating that it can be implemented beyond trial settings to reduce waiting times for patients receiving a range of health services.

## Data Availability Statement

The raw data supporting the conclusions of this article will be made available by the authors, without undue reservation.

## Ethics Statement

The studies involving human participants were reviewed and approved by Eastern Health Human Research Ethics Committee. The participants received written information about the project and implied consent to participate through completion of the online questionnaire.

## Author Contributions

KH and NT led the inception of this project, development of the proposal, provided oversight for implementation of the intervention, and collection of data. KH drafted the manuscript. AL assisted with the project design, implementation of the intervention, collected data, and contributed to the manuscript. DS and BK contributed to the project design, interpretation of findings, and editing of the manuscript. All authors read and approved the manuscript.

## Conflict of Interest

The authors declare that the research was conducted in the absence of any commercial or financial relationships that could be construed as a potential conflict of interest.
